# Classification and pathoanatomy of posterior malleolus fracture based on posterior malleolus-associated ligaments and ankle stability

**DOI:** 10.1016/j.jot.2025.09.003

**Published:** 2025-10-10

**Authors:** Yongqi Li, Yi Liao, Rui Luo, Tian Zhao, Shun Wang, Yunfeng Yang

**Affiliations:** a Department of Orthopedics, Karamay Hospital of People's Hospital of Xinjiang Uygur Autonomous Region (Central Hospital of Karamay), Karamay, 834000, China; b Department of Orthopaedics, Ruijin Hospital, Shanghai Jiao Tong University School of Medicine, Shanghai, 200025, China; c Department of Neurology, Karamay Hospital of People's Hospital of Xinjiang Uygur Autonomous Region (Central Hospital of Karamay), Karamay, 834000, China

**Keywords:** Ankle stability, Classification, Morphology, Pathoanatomy, Posterior malleolus-associated ligaments, Posterior malleolus fracture

## Abstract

**Background:**

The clinical significance of previous posterior malleolus fracture classifications is limited because they are mainly based on fracture morphology. On the basis of posterior malleolus-associated ligaments and ankle stability, injury mechanism, and fracture morphology, a novel posterior malleolus fracture classification system was proposed to clarify the pathoanatomy of posterior malleolus fracture and guide clinical diagnosis and treatment.

**Methods:**

Twenty fresh frozen cadaver specimens of the lower limbs were collected, posterior malleolus-associated ligaments were dissected, and the range of their tibial insertion, average length, and direction were measured. Clinically,we retrospectively analyzed the imaging information of 296 patients with posterior malleolus fractures. Correlating the anatomical measurements of posterior malleolus-associated ligaments of fresh frozen cadaver specimens with the computed tomography(CT) imaging data of posterior malleolus fracture of clinical patients, a clinically practical classification system of posterior malleolus fracture was established. In addition, the novel classification was compared with Haraguci classification and Mason classification.

**Results:**

Posterior malleolus-associated ligaments include the posterior inferior tibiofibular, inferior transverse tibiofibular, and posterior tibiotalar ligaments from the posterolateral to posteromedial tibia. A total of 296 posterior malleolus fractures were divided into three types. Type I posterior malleolus fracture involved only the tibial insertion of the inferior transverse tibiofibular ligament (36 cases, 12.2 %). Type Ⅱ posterior malleolus fracture involved the tibial insertions of the inferior transverse tibiofibular and posterior inferior tibiofibular ligaments and was divided into two subtypes according to whether or not articular cartilage or die-punch injury was present (ⅡA, 150 cases, 50.7 %; ⅡB, 79 cases, 26.7 %). Type Ⅲ posterior malleolus fracture involved all the tibial insertions of the inferior transverse tibiofibular, posterior inferior tibiofibular, and posterior tibiotalar ligaments and included two subtypes according to the fragment number of posterior malleolus fracture (ⅢA, 11 cases, 3.7 %; ⅢB, 20 cases, 6.8 %).

**Conclusions:**

The formation of posterior malleolus fracture stems from the combined effects of external force and internal ligament structure. Correlating posterior malleolus-associated ligaments with the classification of posterior malleolus fracture is of great significance. The proposed classification system considers posterior malleolus-associated ligaments, injury mechanism, and fracture morphology and thus clarifies the pathoanatomy of posterior malleolus fracture and serves as a guide for clinical diagnosis and treatment.

**The translational potential of this article:**

The clinical significance of previous posterior malleolus fracture classifications is limited because they are mainly based on fracture morphology. On the basis of posterior malleolus-associated ligaments and ankle stability, injury mechanism, and fracture morphology, a novel posterior malleolus fracture classification system in our study was proposed. The classification promoted better elucidation of the pathoanatomy of posterior malleolus fracture and guidance for clinical diagnosis and treatment.

## Graphical abstract

**Figure:**
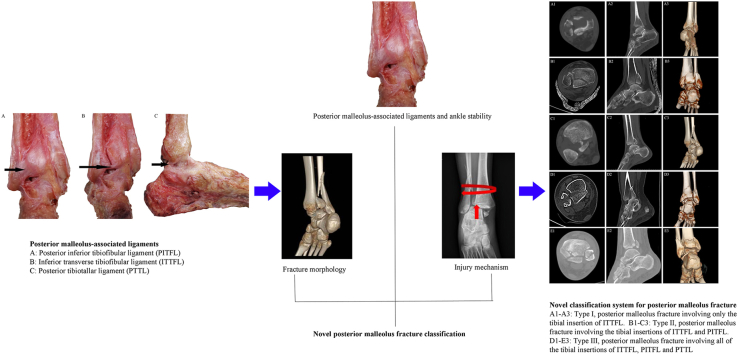

## 1 Introduction

The prognosis of ankle fracture involving the posterior malleolus is relatively poor [[1], [2], [3], [4], [5], [6], [7], [8], [9]]. Posterior pilon fracture is a special type of posterior malleolus fracture, and its prognosis is worse than that of ordinary posterior malleolus fractures due to tending to posterior ankle instability [10]. Treatment options for posterior malleolus fractures with different injury mechanisms and levels of severity and their prognose vary. Many researchers have proposed a variety of classification systems for posterior malleolus fracture to indicate injury mechanism, injury mode, and severity and provide references for treatment and prognosis. However, no consensus has been reached yet. Most classification systems are mainly based on the morphology of posterior malleolus fracture [3,6,10]. Hence, their clinical significance is limited.

With regard to injury mechanism, the formation of posterior malleolus fracture stems from the combined effects of external force and internal ligaments regardless of whether it is a simple rotational-force posterior malleolus fracture or a vertical-rotational-force posterior pilon fracture. We believe that a classification system that considers posterior malleolus-associated ligaments and ankle stability is of great significance. Our research correlated the anatomy of posterior malleolus-associated ligaments with the CT imaging information of clinical patients, and a novel classification system of posterior malleolus fracture that integrated associated ligaments, injury mechanism, and fracture morphology was proposed to clarify the pathoanatomy of the fracture and guide clinical diagnosis and treatment comprehensively and reasonably.

## 2 Materials and methods

This study was performed at Tongji University and Tongji Hospital, and cadaver specimens were obtained from the Department of Anatomy of Tongji University. Twenty fresh frozen cadaver specimens of the lower limbs were used for the dissection of posterior malleolus-associated ligaments. The morphology of each specimen was normal. The clinical and imaging information of 296 patients with posterior malleolus fractures were retrospectively analyzed. The cadaveric anatomy of the tibial insertion of posterior malleolus-associated ligaments was correlated with the CT imaging anatomy of posterior malleolus fractures. Then, each posterior malleolus fracture was classified.

### 2.1 Cadaver dissection and measurement of posterior malleolus-associated ligaments

All 20 specimens were dissected in the same order, and the muscles, tendons, blood vessels, and nerves around the calf and ankle were removed from the distal limb to the proximal limb to fully expose posterior malleolus-associated ligaments. Each of the tibial insertions of posterior malleolus-associated ligaments was identified and measured directly with a digital caliper. Measurement indicators included: the distance between the highest point of tibial insertion of the posterior inferior tibiofibular ligament (PITFL) and the joint line, distance between the highest point of the tibial insertion of the inferior transverse tibiofibular ligament (ITTFL) and the joint line, and the distance between the center of the tibial insertion of the posterior tibiotallar ligament (PTTL) and the intercollicular groove, and the average length of each ligament. The average length of PITFL or ITTFL referred to the averages of the proximal and distal lengths, and the average length of PTTL referred to the averages of anterior and posterior lengths. The angle between PITFL or ITTFL and the horizontal or sagittal plane and the angle between PTTL and the horizontal or coronal plane were measured with a universal protractor. The study was approved by our hospital's internal review board.

### 2.2 Imaging anatomy and measurement of clinical patients

Inclusion criteria: acute posterior malleolus fracture; age ≥18 years; Complete clinical data and preoperative and postoperative imaging information.

Exclusion criteria: old, pathological and open posterior malleolus fracture.

**Fig. 1:**
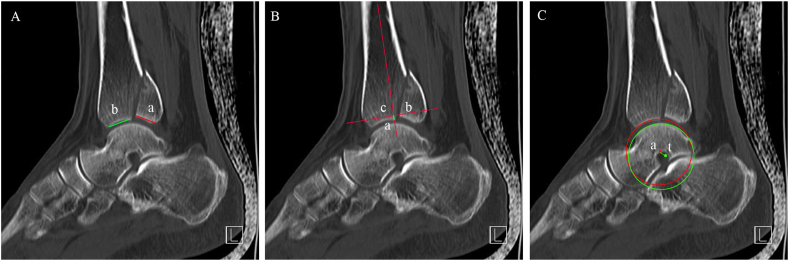
Methods of Lee SH et al. [2] and Marques Ribeiro H et al. [11] were used in measuring the percentage of the articular involvement of the fracture (Figure A: a/(a+b)∗100 %), the extent of posterior talar subluxation (Figure B: distance between a and c), and proximal displacement of posterior malleolus fracture (Figure C: distance between a and t).

From 2012 to 2020, a total of 324 patients with posterior malleolus fractures were admitted. Only 296 of these patients were eligible according to the inclusion and exclusion criteria (224 patients from Shanghai Tongji Hospital and 72 patients from Karamay Central Hospital) (Table 1). The average age was 48.4 years (range, 18–87 years). All patients underwent ankle fluoroscopy (anteroposterior, lateral, and mortise), CT scan (3 mm thickness), and three-dimensional reconstruction upon admission, and ankle fracture and dislocation were examined. The methods of Lee SH et al. [2] and Marques Ribeiro H et al. [11] were used in measuring the percentage of articular involvement of the fracture and the extent of posterior talar subluxation and proximal displacement of posterior malleolus fracture with the largest measurable diameter of the CT sagittal plane (Fig. 1). The cases were anonymized, blinded, and radiographically categorized by three foot and ankle surgeons, and the same patient data was reassessed blindly after six weeks. Interobserver reliability and intraobserver repeatability were assessed using Cohen's kappa coefficient.

**Table 1:** The demographic data of patients with posterior malleolus fractures.

	Number	Percentage
**Gender**		
male	120	40.5 %
female	176	59.5 %
**Left/right**		
left	152	51.4 %
right	144	48.6 %
**The causes of injuries**		
sprain	133	44.9 %
fall	137	46.3 %
car accident	24	8.1 %

### 2.3 Statistical analysis

Statistical Product Service Solutions(SPSS) 25 software was used, and quantitative data(the measurement of posterior malleolus-associated ligaments) were presented as mean and standard deviation (x ± s). First, data were tested for normality with Kolmogorov–Smirnov test and homogeneity of variance with Levene's test. Then, M(Q 1, Q 3)and Mann–Whitney U test was used for data with non-normal distribution(the percentage of articular involvement of the fracture(I), the extent of posterior talar subluxation(S) and the extent of proximal displacement of posterior malleolus fragment(D) between different types). The significance level was set at p < 0.05.

## 3 Results

### 3.1 Anatomy of posterior malleolus-associated ligaments

**Fig. 2:**
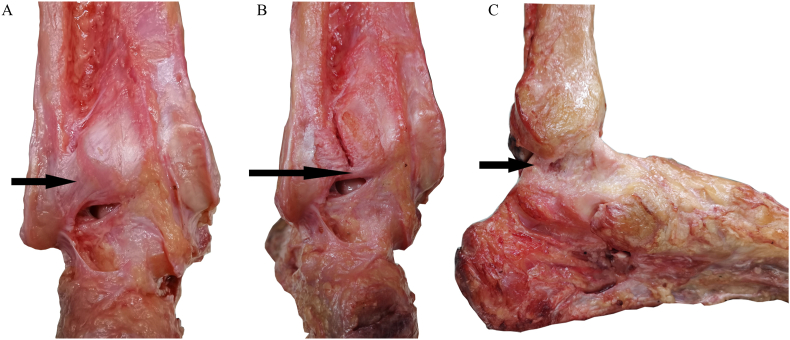
Posterior malleolus-associated ligaments. Figure A: Posterior inferior tibiofibular ligament (PITFL) (arrows) Figure B: Inferior transverse tibiofibular ligament (ITTFL) (arrows) Figure C: Posterior tibiotallar ligament (PTTL) (arrows).

Posterior malleolus-associated ligaments included the PITFL, ITTFL, and PTTL from the posterolateral tibia to the posteromedial tibia (Fig. 2). The PITFL, ITTFL, and deep PTTL were found in all the specimens. The distinguishable superficial PTTL only appeared in 13 specimens. The average length and direction of each ligament and the range of their tibial insertion are shown in Table 2.

**Table 2:** The measurement of posterior malleolus-associated ligaments.

	Range of tibial insertion	Average length (mm)	Direction(°)
PITFL	The PITFL was attached to the posterolateral tibia and the proximal and superficial surfaces of the ITTFL. H_1_:45.2 ± 5.6 mm.	13.3 ± 2.5	Angle_PH_:25.3 ± 3.6 Angle_PS_:82.2 ± 2.8
ITTFL	The ITTFL was attached to the posterior distal tibia. H_2_: 5.5 ± 1.0 mm.	15.2 ± 1.2	Angle_IH_:19.6 ± 3.2 Angle_IS_:85.3 ± 1.6
PTTL	The PTTL was attached to the posterior colliculus and intercollicular groove of the medial malleolus. H_3:_ 2.5 ± 0.6 mm.	14.2 ± 2.1	Angle_PTH_:75.2 ± 3.5 Angle_PTC_:21.1 ± 2.6

PITFL, posterior inferior tibiofibular ligament; ITTFL, inferior transverse tibiofibular ligament; PTTL, posterior tibiotallar ligament.

H_1_, the distance between the highest point of tibial insertion of the PITFL and the joint line; H_2_, the distance between the highest point of the tibial insertion of the ITTFL and the joint line; H_3_, the distance between the center of the tibial insertion of the PTTL and the intercollicular groove.

Angle_PH,_ the angle between PITFL and horizontal plane; Angle_PS,_ the angle between PITFL and sagittal plane; Angle_IH,_ the angle between ITTFL and horizontal plane; Angle_IS,_ the angle between ITTFL and sagittal plane; Angle_PTH,_ the angle between PTTL and horizontal plane; Angle_PTC,_ the angle between PTTL and coronal plane.

### 3.2 Novel classification system for posterior malleolus fracture

**Fig. 3:**
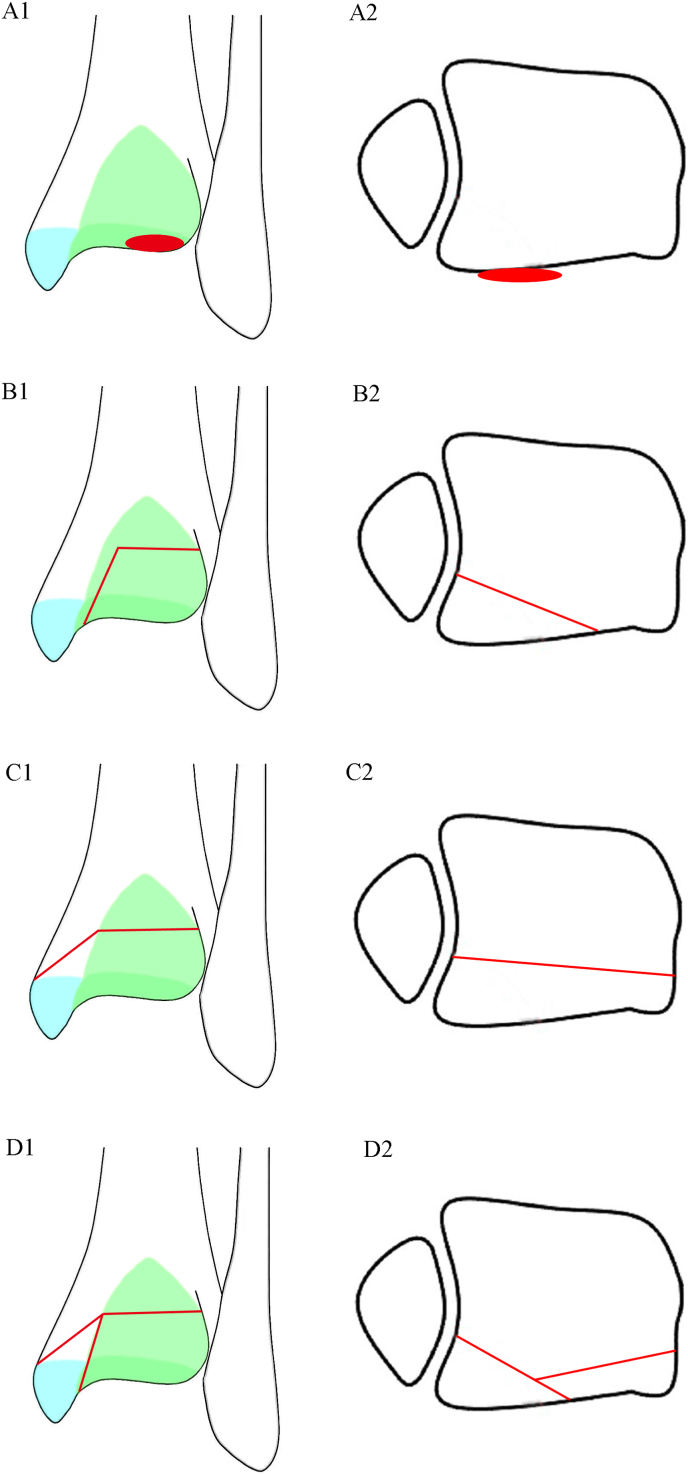
Schematic diagram of novel classification system for posterior malleolus fracture. The dark green, light green, and blue areas represent the tibial insertions of PITFL, ITTFL, and PTTL, respectively. Red represents the posterior malleolar fracture line. A1-A2: Type I, posterior malleolus fracture involving only the tibial insertion of ITTFL; B1-B2: Type Ⅱ, posterior malleolus fracture involving the tibial insertions of ITTFL and PITFL; C1-C2: Type ⅢA, posterior malleolus fracture involving all of the tibial insertions of ITTFL, PITFL and PTTL, and posterior malleolus fracture is a single complete fragment; D1-D2: Type ⅢB, posterior malleolus fracture involving all of the tibial insertions of ITTFL, PITFL and PTTL, and posterior malleolus fracture is divided into two parts: posteromedial and posterolateral fragments.

**Fig. 4:**
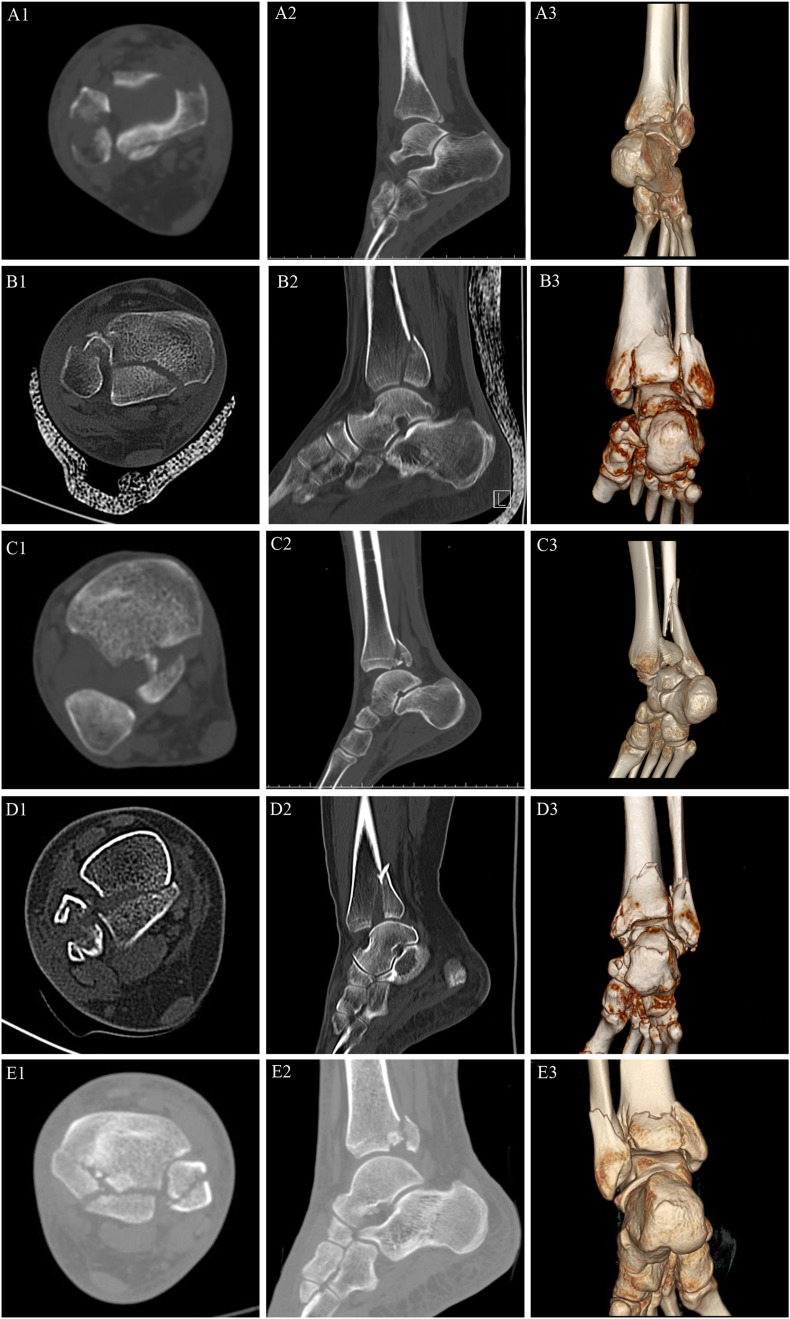
Novel classification system for posterior malleolus fracture. A1-A3: Type I, posterior malleolus fracture involving only the tibial insertion of ITTFL. B1-C3: Type Ⅱ, posterior malleolus fracture involving the tibial insertions of ITTFL and PITFL(B1-B3: TypeⅡA; C1-C3: TypeⅡB). D1-E3: Type Ⅲ, posterior malleolus fracture involving all of the tibial insertions of ITTFL, PITFL and PTTL (D1-D3: Type ⅢA; E1-E3: Type ⅢB).

Based on posterior malleolus-associated ligaments, ankle stability, and injury mechanism and morphology of posterior malleolus fracture, a novel classification system for posterior malleolus fracture was proposed. Using this system, we were able to divide 296 cases into one of the following types:

Type I: posterior malleolus fracture involving only the tibial insertion of the inferior transverse tibiofibular ligament (36 cases; Fig. 3, Fig. 4).

Type Ⅱ: posterior malleolus fracture involving the tibial insertions of inferior transverse tibiofibular and posterior inferior tibiofibular ligaments and was divided into two subtypes according to whether articular cartilage or die-punch injury is present (ⅡA, 150 cases; ⅡB 79 cases; Fig. 3, Fig. 4).

Type Ⅲ: posterior malleolus fracture involving all of the tibial insertions of the inferior transverse tibiofibular, posterior inferior tibiofibular, and posterior tibiotalar ligaments and included two subtypes according to the fragment number of posterior malleolus fracture (ⅢA, 11 cases; ⅢB, 20 cases; Fig. 3, Fig. 4).

In both assessment rounds, the mean Cohen's kappa coefficient for interobserver reliability were 0.969 and 0.971, respectively. In intraobserver repeatability analysis, and the mean Cohen's kappa coefficient was 0.992. Both of the interobserver reliability and intraobserver repeatability were excellent.

Further comparative analysis showed that the percentage of articular involvement of the fracture and the extent of posterior talar subluxation and proximal displacement of the posterior malleolus fractures in type ⅡB fracture were significantly greater than those in type ⅡA fracture. The extent of the posterior talar subluxation and proximal displacement of posterior malleolus fractures in type Ⅲ fracture were significantly greater than in type Ⅱ fracture, (p < 0.01; Table 3).

**Table 3:** Comparison of the percentage of articular involvement of the fracture(I), the extent of posterior talar subluxation(S) and the extent of proximal displacement of posterior malleolus fragment(D) between different types.

		I(%)		S(mm)		D(mm)	
		M(Q 1, Q 3)	*p*	M(Q 1, Q 3)	*p*	M(Q 1, Q 3)	*p*
Novel classify-cation	ⅡA(n = 150)	18. 4(12. 7,21. 7)	<0.01	2. 3(1. 1,3. 0)	<0.01	1. 9(0. 2,3. 0)	<0.01
	ⅡB(n = 79)	23. 7(18. 6,28. 8)		4. 7(1. 5,6. 2)		4. 1(2. 1,6. 0)	
	Ⅱ(n = 229)	—	—	3. 1(1. 1,5. 0)	<0.01	2. 7(0. 4,4. 0)	<0.01
	Ⅲ(n = 31)	—		10. 1(6. 0,15. 0)		7. 2(6. 0,8. 2)	

- No measurement.

## 4 Discussion

Ankle fracture involving posterior malleolus has a poor prognosis [[8], [9], [10], [11], [12], [13]]. A large number of studies have proposed posterior malleolus fracture classification systems, aiming to indicate the mechanism, mode, and severity of the injury and further guide its treatment and determine its prognosis. Current systems for classifying posterior malleolus fracture in the relevant literature mainly include the Haraguchi classification [3], Bartonicek classification [14], Mangnus classification [15], Mason classification [16], AO classification [17], and Lauge-Hansen classification [18]. To date, no consensus has been reached. Current classification systems are mainly based on fracture morphology and do not indicate the injury mechanism and severity. Thus, they have limited significance for treatment and prognosis.

At present, there is still an urgent need for a new clinical practical classification of posterior malleolar fracture to provide references for treatment and prognosis. Combined with the influence of the posterior malleolus-associated ligaments on ankle stability, injury mechanism, and morphology of fracture, we proposed a novel classification system for posterior malleolus fracture, which clarified the pathoanatomy of posterior malleolus fracture and served as a guide for clinical diagnosis and treatment.

### 4.1 Anatomy of posterior malleolus and its influence on the ankle stability

The posterior malleolus maintains ankle stability [19] and plays an important role in the stability of the posterior malleolus [14,20]. In the tibiofibular syndesmosis complex, the PITFL is the most important component that affects the stability of the tibiofibular syndesmosis. The reduction and fixation of posterior malleolus fracture can restore the tension of the PITFL and stabilize syndesmosis [[21], [22], [23], [24]]. In our study, the PITFL was attached to the posterolateral tibia and the proximal and superficial surfaces of the ITTFL, and the tibial insertion was relatively broad. The ITTFL was attached to the posterior distal tibia. The PTTL was attached to the posterior colliculus and intercollicular groove of the medial malleolus, and distinguishing its superficial insertion from a deep one was difficult (Fig. 2).

### 4.2 Novel posterior malleolus fracture classification and its injury mechanism

Based on the morphology and the injury mechanism of posterior malleolar fractures, our study has proposed a novel classification system for posterior malleolar fractures that integrates the influence of the posterior malleolus-associated ligaments on ankle stability, which is innovative to some extent. In posterior malleolus fracture, ankle stability decreases, and the severity of injury increases as the number injured associated ligaments increases. In the proposes system, type Ⅰ is an avulsion fracture of the distal tibia. Theoretically, the injury occurred when the ankle was subjected to only simple rotational-force without vertical-force, and rotational force was relatively small. The pull of the ITTFL resulted in an avulsion fracture of the posterior cortex of the distal tibia. The fragment was small and considered an extra-articular fracture. Type Ⅱ was the posterolateral corner fracture of the tibial plafond without involving the medial malleolus (posterior colliculus). The involved posterior malleolus-associated ligaments included the ITTFL and PITFL. Furthermore, according to different injury mechanisms, type II fractures were further divided into two subtypes. The posterior malleolus fracture without articular cartilage and subchondral bone injury and die-punch fragments was categorized as type ⅡA. Injury was caused by simple rotational force (relatively large) in the ankle. In type ⅡB fracture, the ankle suffers from vertical-rotational compound force, and the posterior malleolus fracture is always accompanied by articular cartilage and subchondral bone injury or die-punch fragments. In type III, posterior malleolus fracture involves the posterior colliculus or intercollicular groove of the medial malleolus and is divided into two subtypes according to the number of fragments. In type ⅢA, posterior malleolus fracture is a single complete fragment vertical force posterior pilon fracture. In type ⅢB, posterior malleolus fracture is divided into two parts: posteromedial and posterolateral fragments and vertical-rotational-force posterior pilon fracture. The mechanism of this injury was surmised to occur with the vertical-rotational compound force in the plantarflexion of the ankle. The involved ligament structures include the ITTFL, PITFL, and PTTL.

Regarding treatment strategies for posterior malleolar fractures: Type I (extra-articular avulsion fractures) may be managed conservatively; Type II warrant surgical fixation via a posterolateral approach (through the interval between the peroneal tendons and flexor hallucis longus) using buttress plates and/or screws; Type III require combined posterolateral and posteromedial approaches (utilizing the intervals between the posteromedial border of the distal tibia and the tibialis posterior tendon) with buttress plate or screw fixation.

Further comparative analysis in this study showed that the percentage of the articular involvement of the fracture and the extent of posterior talar subluxation and proximal displacement of posterior malleolus fractures of the vertical-rotational-force posterior pilon fracture were significantly greater than those of the simple rotational-force posterior malleolus fracture(Type ⅡB VS. type ⅡA, type Ⅲ VS. type Ⅱ, p < 0.01, Table 3).

### 4.3 Advantages of novel posterior malleolus fracture classification

Compared with the previous classification systems, the proposed system overcome various defects and deficiencies, further clarified the pathoanatomy of posterior malleolus fracture [[25], [26], [27], [28]]. In this study, not only the sample size was relatively large but also the fracture types were comprehensive. This classification system clearly distinguished posterior pilon fracture from posterior malleolus fracture: types Ⅰ and ⅡA are simple rotational-force posterior malleolus fracture, types ⅡB and ⅢB are vertical-rotational-force posterior pilon fracture, and type ⅢA is regarded as simple vertical-force posterior pilon fracture. Moreover, the number of injured posterior malleolus-associated ligaments, the range of fracture, and the instability of the posterior ankle progressed as the grade in the proposed increased. Thus, the proposed system may contribute to treatment and prognosis. The results of the comparison between the proposed system and Haraguchi or Mason classification system are shown in Table 4.

**Table 4:** Comparison of novel proposed classification with Haraguchi and Mason classification.

Novel proposed classification	Haraguchi classification	Mason classification	Number	Percentage
Ⅰ	III	Ⅰ	36	12.2 %
ⅡA	Ⅰ	—	150	50.7 %
ⅡB	Ⅰ	ⅡA	79	26.7 %
ⅢA	Ⅱ	Ⅲ	11	3.7 %
ⅢB	Ⅱ	ⅡB	20	6.8 %

- No corresponding fracture type.

The main defects and deficiencies of this study were the limited number of fresh cadaver specimens, insufficient database, and the retrospective design's potential biases (e.g., incomplete follow-up, heterogeneous imaging protocols). They should be further supplemented and improved in the future.

## 5 Conclusions

The new classification system for posterior malleolus fracture, combining posterior malleolus-associated ligaments, fracture injury mechanism, and fracture morphology, not only has more comprehensive fracture types but also clearly distinguishes posterior pilon fracture from posterior malleolus fracture. Meanwhile, the progress of injury severity with the increase in grade of the proposed classification system may provide information that useful in clinical diagnosis and treatment.

## Authors’ contributions

YYF designed the study and provided a critical review of the manuscript. LYQ collected and analyzed the data and wrote the main manuscript. LY and LR provided conceptual advice and the statistical analyses and critically revised the paper. ZT and WS collected the data and prepared Figures and tables. All authors have read and approved the final submitted manuscript.

## Ethics approval and consent to participate

The study was approved by Shanghai Tongji Hospita's internal review board(K-W-2021-015). Documented consent was obtained from the individual who had donated their body or their next of kin, and the body was indeed donated for research or scientific purposes.

## Availability of data and materials

The data sets supporting the conclusion of this article are included in the manuscript. Upon request, raw data can be provided by the corresponding author.

## Funding

This work was supported by 10.13039/100009110Natural Science Foundation of Xinjiang Uygur Autonomous Region(2024D01C11) and Xinjiang Tianshan Talent Training Program (2023TSYCJC0053). YYF took part in the design of the study and collection, analysis, and interpretation of data, and provided a critical review of the manuscript.

## Funding

This work was supported by Natural Science Foundation of Xinjiang Uygur Autonomous Region (2024D01C11) and Xinjiang Tianshan Talent Training Program (2023TSYCJC0053). YYF took part in the design of the study and collection, analysis, and interpretation of data, and provided a critical review of the manuscript.

## Declaration of competing interest

The authors declare that they have no competing interests.
